# Pediatric celiac disease and coexisting immune-mediated conditions: a five-year single-center study from North-Eastern Romania

**DOI:** 10.3389/fped.2026.1786392

**Published:** 2026-06-03

**Authors:** Laura Otilia Boca, Gabriela Ghiga, Gabriela Păduraru, Laura Iulia Bozomitu, Nicoleta Gimiga, Elena Cojocaru, Cătălina Luncă, Elena Lia Spoială, Lorena Mihaela Manole, Laura Mihaela Trandafir

**Affiliations:** 1 Grigore T. Popa University of Medicine and Pharmacy Iasi, Iasi, Romania; 2 Sfanta Maria Emergency Hospital for Children Iasi, Iasi, Romania

**Keywords:** autoimmunity, celiac disease, gluten-free diet, screening, type 1 diabetes mellitus

## Abstract

**Background:**

Celiac disease is among the most common chronic gastrointestinal disorders in childhood and is frequently associated with other autoimmune conditions. The 2020 ESPGHAN guidelines revised pediatric celiac disease diagnosis by allowing non-biopsy confirmation in selected cases. Despite growing global data, evidence from Eastern Europe remains limited, and contemporary regional data describing pediatric celiac disease in routine clinical practice remain scarce.

**Methods:**

We conducted a retrospective, single-center study including 58 pediatric patients with confirmed celiac disease followed between 2020 and 2024 at a tertiary pediatric center in North-Eastern Romania. All patients fulfilled the 2020 ESPGHAN criteria and were stratified according to the presence or absence of coexisting immune-mediated conditions.

**Results:**

Eighteen patients (31.0%) had at least one coexisting immune-mediated condition, most commonly type 1 diabetes mellitus and autoimmune thyroiditis. Female sex predominated in the cohort (female-to-male ratio 3.1:1) and was significantly more common among patients with coexisting immune-mediated conditions. Children with these conditions were diagnosed with celiac disease at an older age (*p* = 0.020) and were more often asymptomatic at diagnosis (27.8% vs. 2.5%; *p* = 0.009). In contrast, children without coexisting immune-mediated conditions were predominantly diagnosed based on symptomatic evaluation, often with mixed gastrointestinal and extraintestinal presentations.

**Conclusions:**

These findings highlight the clinical heterogeneity of pediatric celiac disease according to immune-mediated condition status and suggest that differences in clinical surveillance may influence diagnostic patterns in routine practice. The results support the importance of targeted surveillance and screening strategies in children at increased autoimmune risk.

## 1 Introduction

Celiac disease (CD) is among the most common chronic gastrointestinal disorders in childhood (1). Global estimates indicate a serologic prevalence of approximately 1.4% and a biopsy-confirmed prevalence of roughly 0.7%. In Europe, reported prevalence is around 0.8%, slightly higher than the worldwide biopsy-confirmed rate (2). Over recent decades, the clinical spectrum of CD has expanded beyond classical gastrointestinal manifestations (3, 4). Extraintestinal and silent forms are increasingly recognized, often through screening in at-risk populations (3, 4). The increasing detection of CD reflects improved diagnostic awareness and changes in classification criteria (3). Nevertheless, case detection remains highly dependent on local diagnostic pathways and access to testing. This may result in unequal recognition and delayed diagnosis of pediatric CD across regions.

CD frequently coexists with other autoimmune disorders, particularly type 1 diabetes mellitus (T1DM) and autoimmune thyroiditis. This clustering of autoimmunity likely reflects shared HLA haplotypes and common pathogenic pathways involving loss of immune tolerance (5, 6). In pediatric populations, these comorbidities are clinically relevant, as they may alter disease presentation, influence the timing of diagnosis, and complicate nutritional or metabolic management. Children with T1DM or autoimmune thyroiditis are often followed longitudinally and may undergo periodic serologic screening, increasing the likelihood of identifying asymptomatic or oligosymptomatic CD. In contrast, children without known autoimmune disease are more often diagnosed only after symptom-driven evaluation.

The 2020 ESPGHAN guidelines currently provide the diagnostic framework used in pediatric clinical practice, allowing non-biopsy confirmation in selected cases with high anti-tissue transglutaminase IgA titers [≥10× the upper limit of normal (ULN)] and positive endomysial antibodies (7). These updated criteria changed the diagnostic framework used in pediatric practice and created heterogeneity across studies in terms of diagnostic approach and case ascertainment (8). Importantly, real-world data describing pediatric CD diagnosed under the currently used ESPGHAN framework remain limited.

Pediatric data from Eastern Europe applying the 2020 ESPGHAN criteria are particularly scarce. In Romania, available data indicate persistent underdiagnosis and delayed case recognition, as diagnostic pathways remain largely dependent on symptomatic presentation (9–11). In contrast, several Western and Northern European countries report higher detection rates supported by broader serologic screening initiatives and earlier diagnosis in pediatric cohorts (12–14). These contrasts underscore the need for region-specific epidemiologic assessments. Such data are essential to ensure equitable implementation of diagnostic criteria and to better characterize population-level risk patterns (9, 14).

Given the recent changes in diagnostic criteria and the limited real-world data from Eastern Europe, there is a clear need to characterize current diagnostic patterns of pediatric CD. In this context, several aspects of pediatric CD in Romania remain insufficiently characterized. It remains unclear how children with CD currently present in routine clinical practice, particularly in relation to age at diagnosis, sex distribution, and clinical presentation. Moreover, limited data are available on whether children with coexisting immune-mediated conditions exhibit distinct diagnostic and clinical patterns compared with those with isolated CD. Finally, the interpretation of diagnostic activity after 2020 is challenging, as it coincides with both the implementation of revised guidelines and major disruptions in healthcare access during the COVID-19 pandemic.

This retrospective study aimed to describe the demographic, clinical, serologic, and histologic features of children with CD diagnosed at a tertiary pediatric center in North-Eastern Romania, with a particular focus on coexisting immune-mediated conditions, sex distribution, and age-related diagnostic patterns. While the association between CD and immune-mediated conditions is well established, real-world data from Eastern European pediatric populations remain limited in the context of current diagnostic practice. In this context, the present study provides updated regional data and contributes to a more accurate characterization of pediatric CD in routine clinical practice.

## 2 Materials and methods

We conducted a retrospective, single-center observational study over a five-year period (January 1, 2020—December 31, 2024) at the Sfanta Maria Emergency Hospital for Children in Iași, Romania, a tertiary referral center for pediatric gastroenterology. The study included pediatric patients with CD who were evaluated during hospitalization at our center within the study period. Patients were identified from the institutional electronic database using the International Classification of Diseases (ICD) code corresponding to CD (K90.0). ICD coding was used solely as an initial screening step, after which all potentially eligible cases underwent detailed manual review of medical records, including clinical history, serologic data, and histopathological findings. Only patients with sufficient documentation to confirm that the diagnosis fulfilled the ESPGHAN 2020 criteria were included in the final analysis.

### 2.1 Inclusion criteria

Children aged 0–18 years with confirmed CD who were evaluated during hospitalization at our center between 2020 and 2024 were eligible for inclusion. The final study cohort comprised 58 children who had documented inpatient evaluation during the study period. Of these, 40 were newly diagnosed with CD between 2020 and 2024 and were considered incident cases for the temporal analysis. The remaining 18 patients had been diagnosed before January 1, 2020 but were re-evaluated during hospitalization between 2020 and 2024. These previously diagnosed cases were included only after retrospective verification of diagnostic eligibility and were considered in descriptive and comparative analyses, but were not included as incident cases in temporal analyses.

To ensure diagnostic consistency, the same serological assays were used throughout the study period and were performed in the same institutional laboratory. When available, duodenal biopsy specimens were interpreted by the same pathology team using standardized Marsh classification criteria. No additional histological or serological re-evaluation was performed for cases diagnosed before 2020. Original diagnostic results were used, as testing methods, Marsh classification criteria, and pathology assessment were consistent throughout the study period.

### 2.2 Diagnostic criteria for CD

Diagnostic eligibility was assessed according to the ESPGHAN 2020 criteria for all included patients. For the non-biopsy diagnostic pathway**,** CD was confirmed in children with anti–tissue transglutaminase IgA levels ≥10× ULN, and positive endomysial antibodies. For anti–tissue transglutaminase IgA levels <10× ULN or in cases of selective IgA deficiency, diagnosis required duodenal biopsy showing characteristic lesions (Marsh II–III).

### 2.3 Exclusion criteria

Cases were excluded if they (i) did not meet the 2020 ESPGHAN diagnostic criteria for CD, (ii) had been diagnosed in other centers without accessible documentation, (iii) were incorrectly coded in the database, or (iv) were still undergoing diagnostic evaluation at the time of data extraction.

### 2.4 Patient selection

**Figure 1:**
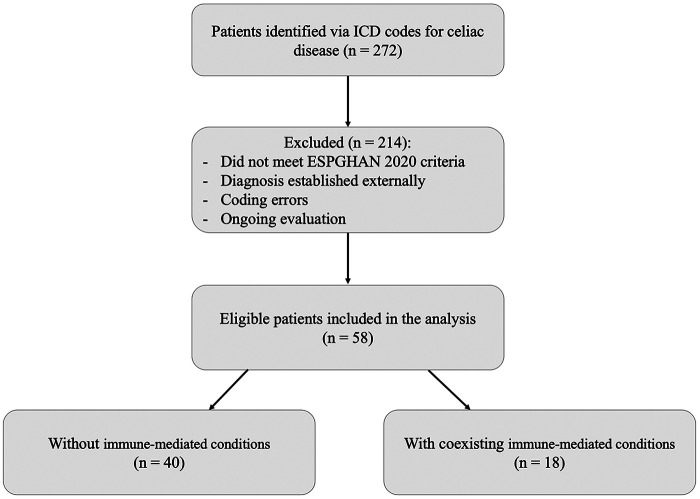
Flowchart illustrating the selection process for the pediatric CD cohort. From an initial pool of 272 patients identified via ICD coding, 214 were excluded based on predefined criteria. The final study cohort comprised 58 children, who were further stratified according to the presence or absence of coexisting immune-mediated conditions.

A total of 272 pediatric cases were initially retrieved. After applying the exclusion criteria, 58 patients with confirmed CD remained eligible for analysis. Among these, 18 children (31%) presented with at least one coexisting immune-mediated condition (Figure 1). One additional patient with CD under surveillance for suspected autoimmune hepatitis was excluded from the immune-mediated subgroup due to diagnostic uncertainty.

### 2.5 Definition of clinical variables

Coexisting immune-mediated conditions were defined as chronic diseases involving immune dysregulation, including both classical autoimmune diseases and immune-mediated conditions with well-established immunological mechanisms. These included T1DM, autoimmune thyroiditis, dermatitis herpetiformis, autoimmune hepatitis, juvenile idiopathic arthritis, systemic lupus erythematosus, and autoimmune hypoparathyroidism. Dermatitis herpetiformis was included in this category as a recognized immune-mediated cutaneous manifestation associated with CD and was retained in order to reflect the overall burden of coexisting immune-mediated pathology at the patient level.

Non-immune-mediated conditions were defined as chronic medical conditions not included in the predefined immune-mediated comparison group and included allergic diseases (e.g., asthma, atopic dermatitis), lactose intolerance, cystic fibrosis, Wilson's disease, and selective IgA deficiency. Although selective IgA deficiency is classified as a primary immunodeficiency and is a recognized immunological association of CD, it was analyzed separately from the subgroup of coexisting immune-mediated conditions because the main comparative framework of the study focused on coexisting autoimmune and immune-mediated diseases rather than primary immunodeficiencies.

Clinical presentations at diagnosis were categorized into three predefined patterns: typical, atypical, and mixed. Typical presentation was defined as gastrointestinal symptoms characteristic of CD, such as chronic diarrhea, abdominal pain, failure to thrive, weight loss, and vomiting. Atypical presentation was defined as predominantly extraintestinal manifestations such as short stature, refractory anemia, cutaneous pallor, persistent elevation of liver enzymes, arthralgia, irritability, or apathy. Mixed presentation was defined as the concomitant occurrence of both gastrointestinal and extraintestinal features. These classifications were based on established descriptions of the clinical spectrum of pediatric CD reported in ESPGHAN guidelines and contemporary review literature (7, 15, 16). Patients presenting with more than one concurrent symptom were considered polysymptomatic, in line with previous pediatric studies (17). Diagnostic delay was defined as the time interval, expressed in years, between age at symptom onset and age at CD diagnosis, and was assessed only in symptomatic patients.

### 2.6 Data collection and analysis

Demographic, clinical, serologic, histologic, and comorbidity data were extracted from electronic medical records. Data entry, descriptive statistics, and all statistical analyses were performed using Microsoft Excel and SPSS Statistics. Continuous variables were expressed as mean ± standard deviation or as median and interquartile range, as appropriate. Normality of continuous variables was assessed using the Shapiro–Wilk test. Because age at CD diagnosis showed a non-normal distribution in one of the subgroups, comparisons between children with and without coexisting immune-mediated conditions were performed using the Mann–Whitney *U*-test. Detailed normality test results and graphical assessments are provided in the Supplementary Materials. Paired comparisons within the same patients were performed using paired *t*-tests when applicable. Categorical variables were analyzed using Fisher's exact test. Temporal trends in the number of newly diagnosed CD cases were initially evaluated using Poisson regression with a log link function. Model fit was assessed using goodness-of-fit statistics, and because overdispersion was identified, a negative binomial regression model was additionally applied as a sensitivity analysis. Detailed goodness-of-fit statistics for the Poisson and negative binomial models are provided in the Supplementary Materials (Supplementary Tables S2, S3). A two-sided *p* value of less than 0.05 was considered statistically significant. No formal correction for multiple testing was applied because the analyses were exploratory and the study was not powered for multiple hypothesis testing.

### 2.7 Overview of epidemiologic and clinical variables analyzed

The epidemiologic variables included sex, age at CD diagnosis, calendar year of diagnosis, and the presence of associated comorbidities, classified as immune-mediated and non-immune-mediated conditions. For patients with coexisting immune-mediated conditions, the specific immune-mediated diagnosis and the number of coexisting immune-mediated conditions were recorded. The clinical variables included mode of presentation at diagnosis (symptomatic or asymptomatic), age at symptom onset, diagnostic delay, type of clinical presentation (typical, atypical, or mixed), symptom burden at diagnosis (number of reported symptoms), and the presence of individual gastrointestinal and extraintestinal manifestations, as documented in the medical records. Serologic and histologic data were collected as part of the diagnostic assessment and included anti–tissue transglutaminase antibodies, endomysial antibodies, total IgA status, and duodenal biopsy findings when available. In the subgroup of children with T1DM, paired metabolic parameters (fasting glucose and HbA1c) before and after initiation of GFD were additionally collected as exploratory descriptive variables.

### 2.8 Ethical approval

The study protocol was reviewed and approved by the Institutional Ethics Committee of Sfanta Maria Emergency Hospital for Children, Iași (number 43096/20.12.2024).

## 3 Results

A total of 58 pediatric patients with confirmed CD were included in the final analysis. The demographic, clinical, and biological characteristics of the study cohort were analyzed with a focus on identifying differences between patients with isolated CD and those with coexisting immune-mediated conditions. Baseline demographic characteristics were compared between children with and without coexisting immune-mediated conditions (Table 1).

**Table 1:** Baseline demographic characteristics of pediatric CD patients according to coexisting immune-mediated condition status.

Variable	Total (*n* = 58)	Without coexisting immune-mediated conditions (*n* = 40)	With coexisting immune-mediated conditions (*n* = 18)	*p*-value
Female sex, *n* (%)	44 (75.9%)	27 (67.5%)	17 (94.4%)	0.046
Male sex, *n* (%)	14 (24.1%)	13 (32.5%)	1 (5.6%)	–
Age at CD diagnosis (years), mean ± SD	5.86 ± 3.50	5.08 ± 3.41	7.26 ± 3.51	0.020
Symptomatic at diagnosis, *n* (%)	52 (89.7%)	39 (97.5%)	13 (72.2%)	0.009
Asymptomatic at diagnosis, *n* (%)	6 (10.3%)	1 (2.5%)	5 (27.8%)	

### 3.1 Associated conditions

**Figure 2:**
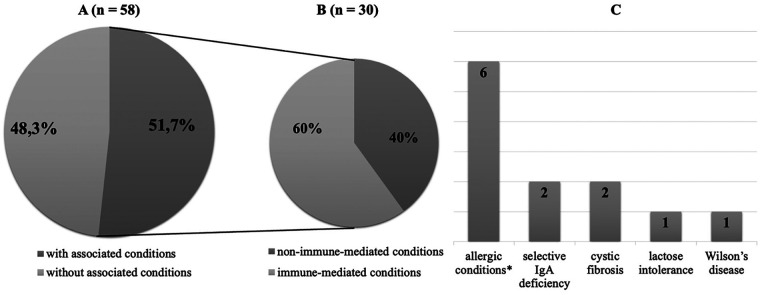
Distribution of associated conditions in the study cohort. **(A)** Proportion of children with and without associated conditions in the total cohort (*n* = 58). **(B)** Distribution of immune-mediated and non-immune-mediated conditions among children with associated conditions (*n* = 30). **(C)** Breakdown of non-immune-mediated conditions. Numeric labels indicate case counts for each category. *Allergic conditions included food-related allergic manifestations (cow's milk protein allergy, egg allergy, and wheat-related allergic manifestations), respiratory and cutaneous allergic disorders (allergic rhinitis, asthma, atopic dermatitis, and urticaria), and sensitization to inhalant allergens.

Of the 58 children included, approximately half of the cohort presented with at least one associated condition (Figure 2). Among associated conditions, immune-mediated conditions predominated over non-immune-mediated ones. Within the non-immune-mediated subgroup, allergic conditions were the most commonly identified and included food-related allergic manifestations (primarily cow's milk protein allergy, egg allergy, and wheat-related allergic manifestations), as well as respiratory and cutaneous allergic disorders such as allergic rhinitis, asthma, atopic dermatitis, urticaria, and sensitization to inhalant allergens. Both IgE-mediated and non-IgE-mediated phenotypes were represented (Figure 2).

**Figure 3:**
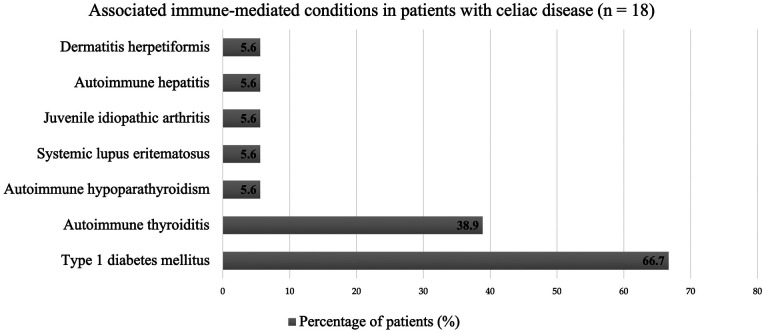
Distribution of immune-mediated conditions associated with CD. Bar chart illustrating the frequency of individual immune-mediated conditions.

Among immune-mediated conditions, T1DM and autoimmune thyroiditis were the most frequently observed conditions, whereas other autoimmune disorders were rare and occurred only sporadically (Figure 3). Six of the 18 children with immune-mediated conditions (33.3%) had three coexisting immune-mediated conditions, including CD. The most frequent combination was CD, T1DM and autoimmune thyroiditis (4/18; 22.2%), whereas rarer associations included CD and T1DM with autoimmune hypoparathyroidism (1/18; 5.6%) and CD and systemic lupus erythematosus with autoimmune hepatitis (1/18; 5.6%). Four of the 18 children with coexisting immune-mediated conditions (22.2%) had a positive family history of autoimmune disorders. Family history included T1DM in two patients and CD in two patients.

### 3.2 Sex distribution and sex-based differences

Among the 58 children included, 44 (75.9%) were girls and 14 (24.1%) were boys, yielding a female-to-male ratio of approximately 3.1:1. Immune-mediated conditions were more frequent among girls: 17 of the 18 children (94.4%) with coexisting immune-mediated conditions were female, while only one male patient (5.6%) exhibited an immune-mediated condition (Table 1). The association between female sex and the presence of immune-mediated conditions was statistically significant (*p* = 0.046, Fisher's exact test). Among children without coexisting immune-mediated conditions, the female predominance was less pronounced (Table 1).

### 3.3 Age at diagnosis and temporal associations

Of the total cohort, 52 (89.7%) were symptomatic at the time of diagnosis. The mean age at diagnosis for the entire cohort (symptomatic and asymptomatic patients) was 5.86 years. Among symptomatic patients (*n* = 52), the mean age at symptom onset was 3.5 years, and the mean age at diagnosis was 5.2 years. Diagnostic delay showed a markedly skewed distribution and was therefore summarized using the median and interquartile range (IQR). The median diagnostic delay was 0 years (IQR 0–1), with a mean delay of 0.93 years.

**Figure 4:**
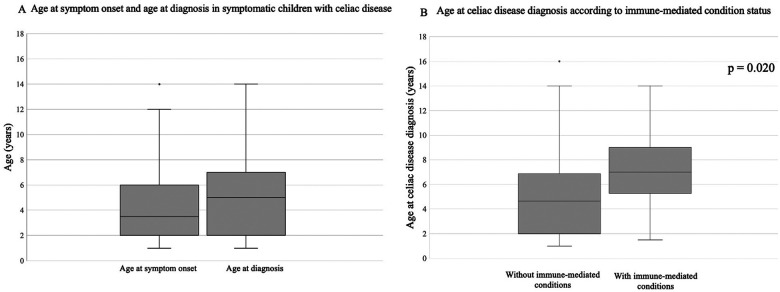
Diagnostic timing and age distribution in pediatric CD. **(A)** Age at symptom onset and age at diagnosis in symptomatic children with CD. Boxplots illustrate the distribution of age at symptom onset and age at diagnosis. **(B)** Age at CD diagnosis according to immune-mediated condition status. Children with coexisting immune-mediated conditions were diagnosed at a significantly older age compared with those without such conditions (median values; Mann–Whitney *U*-test, U = 222.5, *p* = 0.020).

Most symptomatic children (80%) were diagnosed within one year of symptom onset (Figure 4), although a small number exhibited prolonged delays of up to 10 years. The presence of several extreme values further supported the use of non-parametric summary measures.

Children with immune-mediated conditions were diagnosed at an older age compared to those without such conditions (mean ± SD: 7.26 ± 3.51 vs. 5.08 ± 3.41 years). This difference was statistically significant (Mann–Whitney *U*-test, U = 222.5, *p* = 0.020) (Figure 4). A non-parametric test was used, as Shapiro–Wilk analysis indicated a non-normal distribution of age at diagnosis in the non-immune-mediated group. This analysis excluded one case with CD and uncertain immune-mediated condition status, who was under evaluation for possible autoimmune hepatitis. In univariate analysis, female sex and older age at diagnosis were significantly associated with the presence of immune-mediated conditions. A multivariate logistic regression model was not performed due to the limited sample size.

**Figure 5:**
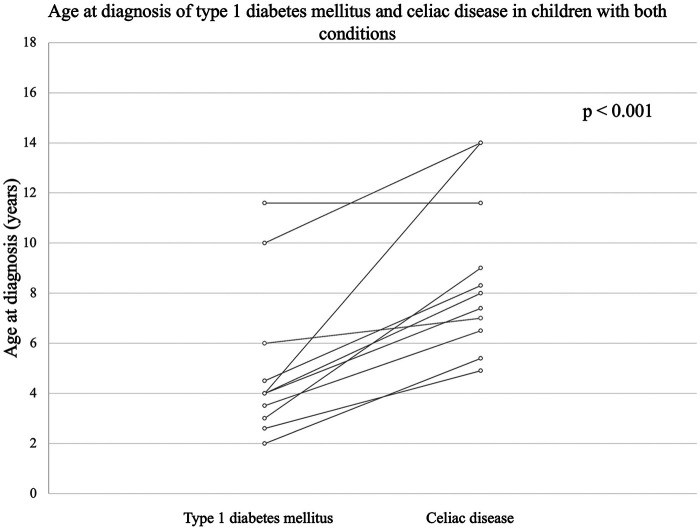
Age at diagnosis of T1DM and CD in children with both conditions (*n* = 12). Paired *t*-test showed that T1DM was diagnosed significantly earlier than CD.

In the subgroup of patients diagnosed with both T1DM and CD (*n* = 12), we analyzed the temporal relationship between the two diagnoses. The mean age at onset of T1DM was 5.10 ± 2.93 years, significantly earlier than the diagnosis of CD, which occurred at a mean age of 8.59 ± 3.06 years (*p* < 0.001, paired *t*-test) (Figure 5). A moderate positive correlation was observed between the age at T1DM diagnosis and the age at CD diagnosis (Pearson's r = 0.62).

### 3.4 Clinical presentation and symptom burden

The clinical presentation at CD diagnosis differed between children with and without coexisting immune-mediated conditions. As shown in Table 2, asymptomatic presentation was significantly more frequent in children with immune-mediated conditions (*p* = 0.009). When symptomatic, these patients more often exhibited a typical presentation, with fewer mixed or exclusively atypical presentations. The overall distribution of symptom counts did not differ significantly between groups (*χ*² = 5.33, *p* = 0.149), although the comparison of ≤2 vs. >2 symptoms showed a borderline association (*p* = 0.055). Detailed frequencies are presented in Table 2.

**Table 2:** Distribution of symptom burden at diagnosis among pediatric CD patients, according to the presence of immune-mediated conditions.

Symptoms	Total, *n* (%)	Without immune-mediated conditions *n* (%)	With immune-mediated conditions *n* (%)	*p*-value
Asymptomatic at diagnosis	6 (10.3%)	1 (2.5%)	5 (27.8%)	0.009
Symptomatic at diagnosis	52 (89.7%)	39 (97.5%)	13 (72.2%)	
1 symptom	17 (32.6%)	10 (25.6%)	7 (53.8%)	
2 symptoms	10 (19.2%)	7 (17.9%)	3 (23%)	
3 symptoms	10 (19.2%)	8 (20.5%)	2 (15.3%)	
>3 symptoms at diagnosis	15 (28.8%)	14 (35.8%)	1 (7.7%)	
≤2 vs. >2 symptoms	–	–	–	0.055

In contrast, children without coexisting immune-mediated conditions were rarely asymptomatic at diagnosis. They were predominantly identified based on symptomatic presentations, most commonly with mixed gastrointestinal and extraintestinal features. Individual extraintestinal manifestations are summarized in Table 4. In four children, elevated transaminases were attributed to alternative diagnoses (hypothyroidism, Wilson disease, cystic fibrosis, or ongoing evaluation for seronegative autoimmune hepatitis) rather than to CD.

#### 3.4.1 Gastrointestinal manifestations

Gastrointestinal symptoms were the predominant mode of presentation. Among symptomatic patients with CD, the most frequent gastrointestinal manifestations were failure to thrive (59.6%), abdominal pain (38.4%), and diarrhea (26.9%). The prevalence of each gastrointestinal manifestation in the two subgroups is shown in Table 3. None of the differences reached statistical significance (*p* > 0.050 for all comparisons).

**Table 3:** Distribution of gastrointestinal symptoms at diagnosis in pediatric CD, according to immune-mediated condition status.

Gastrointestinal symptoms	Total, *n* (%)	Without immune-mediated conditions *n* (%)	With immune-mediated conditions *n* (%)	*p*-value
Failure to thrive	31 (59.6%)	25 (64.1%)	6 (46.1%)	0.332
Abdominal pain	20 (38.4%)	18 (46.1%)	2 (15.3%)	0.057
Diarrhea	14 (26.9%)	12 (30.7%)	2 (15.3%)	0.472
Decreased appetite	11 (21.1%)	9 (23%)	2 (15.3%)	0.709
Abdominal distension	9 (17.3%)	6 (15.3%)	3 (23%)	0.674
Constipation	8 (15.3%)	7 (17.9%)	1 (7.6%)	0.662
Undigested stools	6 (11.5%)	5 (12.8%)	1 (7.6%)	1.000
Bulky stools	4 (7.6%)	3 (7.6%)	1 (7.6%)	1.000
Vomiting	3 (5.7%)	2 (5%)	1 (7.6%)	1.000
Food refusal	3 (5.7%)	3 (7.6%)	0	0.564
Intestinal intussusception	2 (3.8%)	1 (2.5%)	1 (7.6%)	0.441
Alternating diarrhea and constipation	1 (1.9%)	1 (2.5%)	0	1.000

#### 3.4.2 Extraintestinal manifestations

Extraintestinal manifestations were overall less frequent compared to gastrointestinal symptoms. The most common findings were cutaneous pallor (28.8%) and short stature (23.0%), followed by persistent elevated transaminases (13.4%), refractory anemia (11.5%), irritability/apathy (9.6%), and arthralgia (5.7%). No statistically significant differences were found between patients with and without immune-mediated conditions for any of the analyzed extraintestinal parameters (*p* > 0.050 for all comparisons). The detailed distribution is shown in Table 4.

**Table 4:** Distribution of extraintestinal symptoms at diagnosis in pediatric CD, according to immune-mediated condition status.

Extraintestinal symptoms	Total, *n* (%)	Without immune-mediated conditions *n* (%)	With immune-mediated conditions *n* (%)	*p*-value
Cutaneous pallor	15 15 (28.8%)	12 (30.7%)	3 (23%)	0.733
Short stature	12 12 (23.0%)	10 (25.6%)	2 (15.3%)	0.706
Elevated transaminases	7 7 (13.4%)	5 (12.8%)	2 (15.3%)	1.000
Refractory anemia	6 6 (11.5%)	4 (10.2%)	2 (15.3%)	0.632
Irritability/apathy	5 5 (9.6%)	4 (10.2%)	1 (7.6%)	1.000
Arthralgia	3 (5.7%)	2 (5.1%)	1 (7.6%)	1.000

#### 3.4.3 Distribution of presentation types

When comparing the clinical presentation types among symptomatic patients, those with immune-mediated conditions showed a higher proportion of typical presentations (53.8% vs. 32.4%), whereas mixed presentations predominated in the group without immune-mediated conditions (59.5% vs. 23.1%). The overall distribution of presentation types showed a borderline statistical difference (*χ*² = 5.85, *p* = 0.054), while the comparison of typical vs. atypical presentations did not reach statistical significance (*p* = 0.192).

Exploratory inspection of symptom counts by sex did not reveal statistically interpretable differences due to the very small number of male patients in both subgroups. Therefore, sex-based comparisons were not included in the analysis.

### 3.5 Serological and histological findings

All patients included in the study were diagnosed with CD according to the 2020 ESPGHAN guidelines**,** using a combination of serological markers, endomysial antibody testing, and histopathological confirmation when applicable.

**Figure 6:**
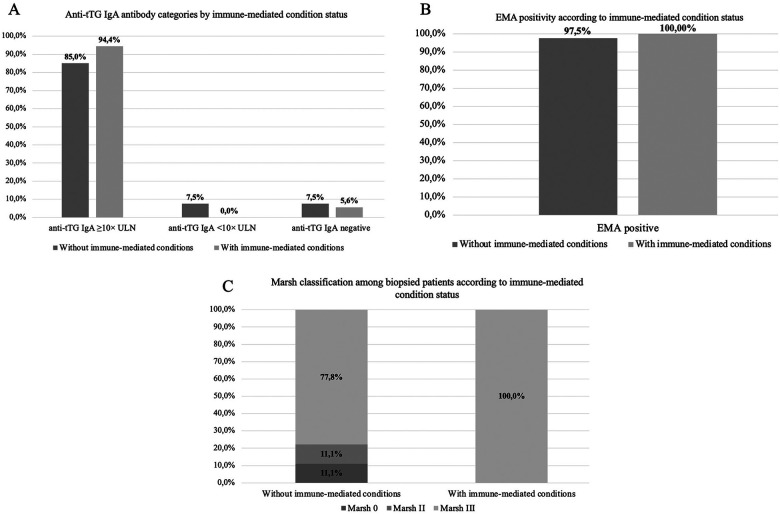
Serologic and histologic characteristics according to immune-mediated condition status. **(A)** Distribution of anti-tTG IgA antibody categories by immune-mediated condition status. For graphical purposes, patients with selective IgA deficiency were included in the anti-tTG IgA negative category. This category included two patients without immune-mediated conditions and one patient with immune-mediated conditions who had selective IgA deficiency and were diagnosed based on characteristic duodenal biopsy findings. **(B)** EMA positivity according to immune-mediated condition status. **(C)** Marsh classification among patients undergoing duodenal biopsy according to immune-mediated condition status. Percentages for Marsh categories were calculated only among biopsied patients.

Among the 18 children with CD and coexisting immune-mediated conditions, 17 (94.4%) had anti-tissue transglutaminase IgA antibody levels ≥10× ULN. All of these patients also tested positive for anti-endomysial antibodies. One patient with selective IgA deficiency had negative anti-tTG IgA antibodies and was diagnosed based on elevated anti-tissue transglutaminase IgG antibodies and characteristic duodenal biopsy findings. Histological examination was performed in six patients, all of whom exhibited Marsh grade III lesions (A–C), confirming villous atrophy (Figure 6).

In the group without coexisting immune-mediated conditions (*n* = 40), 34 patients (85.0%) had anti-tissue transglutaminase IgA antibody levels ≥10× ULN, while 3 had titers below this threshold and 3 had a negative result. Two patients with selective IgA deficiency had negative anti-tTG IgA and were diagnosed based on characteristic duodenal biopsy findings. A total of 39 (97.5%) were anti-endomysial antibodies-positive. Duodenal biopsy was performed in 9 cases: 1 had normal histology (Marsh 0, biopsy performed at parental request despite meeting serologic criteria for non-biopsy diagnosis), while 1 showed Marsh II and 7 showed Marsh III lesions (A-C). The proportion of children with anti-tissue transglutaminase IgA antibody titers ≥10× ULN did not differ significantly between the two groups (*p* = 0.417). Detailed subgroup distributions of anti–tissue transglutaminase antibody categories, endomysial antibody status, and Marsh classification are additionally provided in Figure 6.

**Figure 7:**
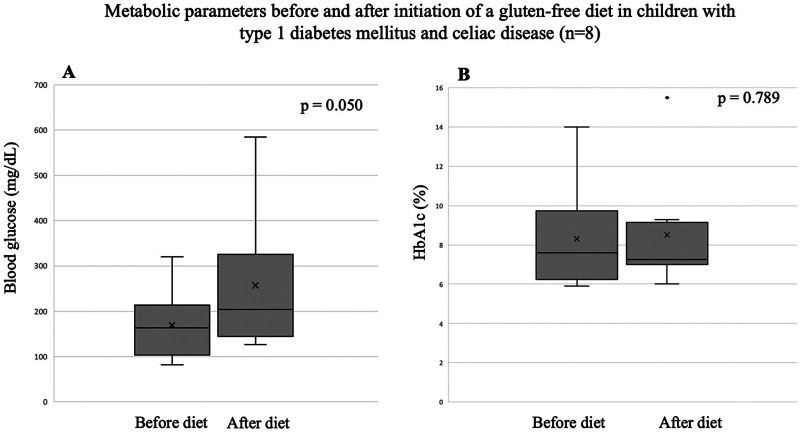
Metabolic parameters before and after initiation of GFD in pediatric patients with T1DM and CD (*n* = 8). **(A)** Fasting blood glucose levels (mg/dL) before and after GFD. **(B)** HbA1c (%) before and after GFD. Boxplots represent the median and interquartile range, and whiskers indicate the full range of values. Mean values are shown as “x” markers. Exact paired *t*-test *p*-values are indicated above each panel.

In an exploratory subgroup analysis of eight pediatric patients diagnosed with both T1DM and CD, paired glycemic data were available before GFD initiation and after a follow-up interval of 3–6 months. As shown in Figure 7, mean fasting glucose increased from 169.6 mg/dL to 256.9 mg/dL (mean difference +87.3 mg/dL; *p* = 0.050), whereas mean HbA1c remained stable (8.32% to 8.50%; mean difference +0.18%; *p* = 0.789).

### 3.6 Temporal distribution of diagnoses

Among the 58 pediatric patients included in the study, 40 received their initial diagnosis of CD between 2020 and 2024, while 18 had been diagnosed before 2020 and were re-evaluated or followed in our center during the study period. Only newly diagnosed cases between 2020 and 2024 were included in the temporal trend analysis. The annual distribution of new diagnoses was as follows: 1 case in 2020, 8 cases in 2021, 2 cases in 2022, 6 cases in 2023, and 23 cases in 2024. Poisson regression suggested an increasing trend in the number of newly diagnosed CD cases over time (*p* < 0.001). However, goodness-of-fit statistics indicated substantial overdispersion (Pearson *χ*²/df > 4). To account for this, a negative binomial regression model was applied as a sensitivity analysis. In this model, the temporal trend was no longer statistically significant (*p* = 0.12).

When diagnoses were grouped according to timing (before vs. after 2020), children with immune-mediated conditions were more frequently diagnosed with CD prior to 2020 compared with those without such conditions (61.1% vs. 20.0%; Fisher's exact test, *p* = 0.005). Patients diagnosed before 2020 were included only in descriptive and comparative analyses and were not considered incident cases during the study period.

## 4 Discussion

The temporal distribution of CD diagnoses in our cohort between 2020 and 2024 should be interpreted cautiously, in the context of evolving referral patterns and changes in healthcare utilization. Although crude Poisson regression suggested an increasing trend in newly diagnosed cases, this finding was not confirmed after accounting for overdispersion using a negative binomial model, indicating that the observed pattern was not robust across modeling approaches. Rather than reflecting a true increase in disease frequency, the annual variation in diagnoses may have been influenced by factors unrelated to disease epidemiology. In particular, the low number of diagnoses observed in 2020–2021 may reflect reduced healthcare utilization during the COVID-19 pandemic, followed by recovery in subsequent years. Taken together, these findings suggest that fluctuations in diagnosis rates may primarily reflect variation in referral patterns and case ascertainment rather than a true epidemiologic shift.

The association between CD and coexisting immune-mediated conditions is well recognized in the literature. Therefore, the contribution of the present study does not lie in demonstrating this relationship *per se*, but in describing clinical presentation, diagnostic pathways, and comorbidity-related differences in a real-world pediatric cohort from North-Eastern Romania under the ESPGHAN 2020 diagnostic framework.

Several differences were observed between children with and without immune-mediated conditions in our cohort. Children in this subgroup were more frequently diagnosed prior to 2020. This pattern may reflect earlier referral or increased clinical surveillance in this subgroup, although our study design does not allow determination of causality. They were also older at diagnosis and more often asymptomatic, consistent with the fact that many undergo periodic testing as part of routine follow-up for their underlying conditions.

In our cohort, a subset of children presented with multiple coexisting immune-mediated conditions, with 6 out of 18 patients exhibiting more than one associated condition. Among children with multiple immune-mediated conditions, the most frequent combination involved T1DM and autoimmune thyroiditis. In addition, four children with coexisting immune-mediated conditions had a family history of autoimmune disorders, including CD, further suggesting the possibility of a broader susceptibility background in this subgroup. Although our sample size does not allow formal characterization of specific polyautoimmune phenotypes, these observations are consistent with the concept of polyautoimmunity, in which distinct immune-mediated disorders coexist within the same individual (18, 19).

Similar patterns of autoimmune clustering have also been described in other CD populations, where multiple autoimmune comorbidities were frequently observed (20). Previous studies have suggested that autoimmune clustering in CD may reflect broader susceptibility mechanisms involving both genetic and immunologic factors rather than isolated disease associations. Shared HLA haplotypes, particularly HLA-DQ2 and HLA-DQ8, represent an important genetic link between CD and several autoimmune disorders, especially T1DM (18, 19). Additional mechanisms involving altered immune regulation and impaired immune tolerance have also been proposed.

More recently, pediatric studies investigating autoantibody signatures in children with polyautoimmunity have suggested that broader patterns of immune dysregulation may accompany autoimmune clustering, further supporting the concept of shared susceptibility across immune-mediated conditions (21). However, no genetic or mechanistic analyses were performed in our study, and these considerations should therefore be interpreted only as potential explanatory frameworks derived from previous literature.

From a clinical perspective, the coexistence of multiple immune-mediated disorders in pediatric patients has important implications for long-term surveillance and multidisciplinary management. The presence of one autoimmune condition may increase the likelihood of detecting additional associated disorders over time, either through shared pathogenic mechanisms or through intensified clinical monitoring. In this context, children with CD and coexisting immune-mediated conditions may benefit from continued surveillance and periodic screening for associated autoimmune diseases, particularly autoimmune thyroiditis and T1DM, even in the absence of overt symptoms. Our findings are aligned with current recommendations supporting surveillance for associated autoimmune conditions in children with CD and reinforce the importance of maintaining a high index of suspicion for additional immune-mediated conditions in pediatric populations considered at increased autoimmune risk.

In contrast, children without immune-mediated conditions were predominantly diagnosed on the basis of gastrointestinal or mixed clinical presentations. Therefore, the temporal differences observed between groups may reflect differences in clinical surveillance and referral patterns. It is also possible that children with immune-mediated conditions more frequently meet serologic criteria that prompt evaluation for CD. Taken together, these findings are compatible with a diagnostic trajectory in which CD associated with immune-mediated conditions is more often detected through targeted testing, whereas CD in children without immune-mediated conditions remains largely symptom-driven.

Allergic conditions represented the most frequent non-immune-mediated associated conditions in our cohort and comprised a heterogeneous spectrum of food-related manifestations as well as respiratory and cutaneous allergic disorders. Several children exhibited multiple concomitant allergic conditions, including combinations of cow's milk protein allergy, egg allergy, wheat-related allergic manifestations, allergic rhinitis, atopic dermatitis, asthma, and urticaria. Food-related manifestations included both IgE-mediated and non-IgE-mediated forms. From a clinical perspective, these observations may be relevant because food-related allergic disorders and CD can present with partially overlapping manifestations during childhood. Symptoms such as abdominal pain, diarrhea, vomiting, feeding difficulties, growth impairment, and failure to thrive may occur in both conditions and may complicate differential diagnosis, particularly in younger children with nonspecific presentations. In routine pediatric practice, these symptoms may initially be attributed to allergic disorders, potentially delaying recognition of underlying CD in some patients.

Previous reports have suggested that food allergies and CD may coexist despite distinct pathogenic mechanisms and immunologic pathways (22). In our cohort, the presence of multiple concomitant allergic conditions in several children further illustrates the clinical complexity that may accompany pediatric CD. However, current evidence does not strongly support a major shared biological background. Recent Mendelian randomization analyses investigating associations between CD and type 2 inflammatory conditions such as asthma, allergic rhinitis, atopic dermatitis, and IgE-mediated food allergy identified statistically detectable associations, although their magnitude appeared limited and of uncertain clinical relevance (23). Taken together, these observations suggest that overlap encountered in pediatric practice may be more important from a clinical and diagnostic perspective than as evidence of a strong shared genetic susceptibility.

In our cohort, a female predominance was observed, with a female-to-male ratio of approximately 3.1:1. This finding is consistent with previous epidemiologic studies reporting higher prevalence of pediatric CD in girls, with female-to-male ratios generally ranging from 2:1 to 3:1 (6). A similar female predominance has also been previously reported across pediatric autoimmune disorders more broadly, where females are affected more frequently than males (24, 25). Although the sex ratio observed in our cohort was slightly higher than that reported in some studies, this difference may reflect sample size, referral patterns, or population-specific characteristics.

In the analyzed cohort, sex disparity was more pronounced in the subgroup with coexisting immune-mediated conditions, where 94.4% of affected children were female, resulting in a significant association between female sex and the presence of immune-mediated conditions. This finding is broadly in line with epidemiologic observations showing a higher prevalence of autoimmune diseases in girls. However, this observation should be interpreted in the context of the limited number of male patients in our cohort. Larger, multicenter studies are needed to determine whether this pattern reflects true sex-related differences in risk or is influenced by sample size, referral patterns, or other population-specific factors.

In the present study, children with immune-mediated conditions were diagnosed with CD at a significantly older age compared with those without such conditions. This difference is consistent with the higher proportion of asymptomatic presentations in this subgroup. It may reflect routine serologic screening performed during follow-up for underlying immune-mediated conditions. In contrast, children without immune-mediated conditions were more often diagnosed through symptom-driven evaluation, which may result in earlier clinical recognition.

In our cohort of children diagnosed with both T1DM and CD, T1DM was identified at a significantly younger age than CD, with a mean interval of approximately three years between the two diagnoses. A moderate positive correlation was observed between the age at onset of T1DM and the age at diagnosis of CD, indicating that children diagnosed later with T1DM also tended to be diagnosed later with CD. These findings describe the diagnostic sequence observed in our study population and reflect local clinical practice and screening patterns.

To place these results in a broader context, similar temporal relationships between T1DM and CD have been reported in previous pediatric studies. Sahin et al. showed that most cases of CD in children with T1DM were detected within the first five years of diabetes follow-up (26). Similarly, Slae et al. and Pham-Short et al. reported that the risk of CD remains highest at the time of T1DM diagnosis and during the early years thereafter (27, 28).

In comparison with these studies (27, 28), the interval between T1DM onset and CD diagnosis in our cohort appeared slightly longer. This may reflect differences in local screening practices or clinical thresholds for testing. Nevertheless, the three-year mean interval between diagnoses in our population remains consistent with current recommendations for periodic CD screening in children with T1DM.

Clinical presentation differed substantially according to immune-mediated condition status in our cohort. More than one quarter of children with immune-mediated conditions were asymptomatic at the time of CD diagnosis, whereas asymptomatic cases were rare among children without such conditions. When symptomatic, children with immune-mediated conditions more frequently presented with typical presentations, while mixed presentations predominated in children without immune-mediated conditions. These patterns reflect the clinical presentation observed in our study population and suggest distinct diagnostic pathways according to immune-mediated condition status.

Comparable findings have been described in previous screening-based studies and pediatric cohorts. For example, Tommasini et al. reported that approximately two-thirds of children identified through a school-based serologic screening program were clinically silent at diagnosis (29). Similarly, studies in pediatric T1DM populations have shown that many children with biopsy-confirmed CD are asymptomatic at diagnosis, supporting the well-recognized silent presentation of CD in the context of coexisting autoimmunity (28, 30).

Symptom burden in our cohort followed a similar pattern. Children with immune-mediated conditions more often presented with single or few symptoms, whereas multisystemic or mixed gastrointestinal and extraintestinal manifestations predominated among children without such conditions. Gastrointestinal symptoms such as abdominal pain and growth failure were more prevalent in the non-immune-mediated group, although none of the individual gastrointestinal manifestations reached statistical significance. Overall, these findings suggest a milder or less typical clinical profile in immune-mediated CD.

Similar observations have been reported in previous pediatric studies. Dolinšek et al. found that 47.6% of symptomatic children with CD were polysymptomatic at diagnosis (17). In our cohort, this concept was reflected by the analysis of symptom burden, with multiple concurrent symptoms occurring more frequently in children without immune-mediated conditions, whereas children with immune-mediated conditions were more often asymptomatic or oligosymptomatic. This finding indicates that polysymptomatic presentations are common in pediatric CD, particularly among children diagnosed following symptom-driven evaluation.

This difference in symptom burden is consistent with distinct diagnostic pathways. In children with immune-mediated conditions, CD is frequently identified through targeted serologic screening during routine follow-up, leading to earlier or asymptomatic detection. In contrast, children without immune-mediated conditions tend to be diagnosed when symptoms develop, often with more complex or multisystemic presentations. The wide variability of clinical manifestations in our cohort, from entirely silent cases identified through surveillance to highly symptomatic children, underscores the heterogeneity of CD presentation in childhood. These findings support current recommendations for systematic screening in pediatric populations at increased risk.

All children in our cohort fulfilled the 2020 ESPGHAN diagnostic criteria. Because these guidelines allow a non-biopsy diagnosis in many cases, histologic assessment was available only for a limited number of duodenal biopsies. In this context, previous data from a Romanian pediatric cohort reported by Belei et al. showed that mucosal normalization under a GFD generally occurs within 1–3 years, while persistent villous atrophy was observed mainly in children with selective IgA deficiency (31).

In an exploratory analysis of a small subgroup of children with both T1DM and CD, we observed an increase in mean fasting glucose levels after initiation of a GFD, showing a borderline trend. In contrast, HbA1c levels remained clinically stable during follow-up. Similar findings have been reported in other pediatric cohorts, in which HbA1c showed little change during early follow-up despite increases in insulin requirements or fasting glucose levels. This phenomenon has been attributed by some authors to improved intestinal absorption after mucosal recovery (32). However, because detailed data on insulin dose adjustments were not available in our cohort, changes in fasting glucose levels cannot be attributed solely to dietary intervention. Conversely, other studies have reported modest improvements in HbA1c after longer periods of strict dietary adherence, suggesting that metabolic responses to a GFD may vary according to population characteristics and follow-up duration (33). Taken together, our observations are consistent with the heterogeneity described in the literature and underline the importance of close metabolic monitoring during the initial months of dietary transition in children with T1DM and CD.

## 5 Limitations

This study has several limitations that should be acknowledged. First, the overall sample size was relatively small. In particular, the very low number of male patients with immune-mediated conditions reduced the statistical power of subgroup analyses, limiting the stability of some associations. As this was a retrospective, single-center study, the sample size was determined by case availability rather than by *a priori* calculation, and no formal statistical power analysis was performed. In addition, we acknowledge that dermatitis herpetiformis may also be considered part of the CD spectrum rather than a separate comorbidity. However, it was retained within the broader category of coexisting immune-mediated conditions in order to reflect the overall burden of associated immune-mediated pathology in our cohort.

Second, metabolic follow-up after initiation of the GFD was available only for a small subgroup of children with T1DM. The follow-up interval ranged from 3 to 6 months, and detailed information on insulin regimen adjustments during this period was not available. These factors may have influenced metabolic outcomes, limited statistical power, and restricted the ability to draw firm conclusions regarding short-term glycemic changes. Therefore, these findings should be interpreted with caution and considered exploratory.

Finally, the retrospective single-center design carries inherent risks of selection bias, incomplete documentation, and limited generalizability. In addition, because the cohort was derived from a single tertiary center and included only hospitalized patients evaluated at our institution, the findings may also reflect local referral patterns and case mix. These factors may limit the external validity of our findings and their applicability to broader pediatric populations. Larger prospective, multicenter studies are needed to confirm these findings and to further define the clinical characteristics of pediatric CD with immune-mediated conditions.

Despite these limitations, the study provides useful descriptive data on the clinical presentation and diagnostic patterns of pediatric CD in a tertiary referral center, contributing to the limited evidence available from this region.

## 6 Conclusions

This study highlights the clinical heterogeneity of pediatric CD in relation to coexisting immune-mediated conditions and suggests that differences in diagnostic pathways may influence how affected children are identified in routine clinical practice. The findings support the importance of targeted surveillance in children at increased autoimmune risk while also reinforcing the need for heightened clinical awareness in children without known immune-mediated conditions, in whom diagnosis remains largely symptom-driven. In the context of limited pediatric data from Eastern Europe, these results provide contemporary real-world evidence and contribute to a more comprehensive characterization of pediatric CD in current clinical practice.

## Data Availability

The raw data supporting the conclusions of this article will be made available by the authors, without undue reservation.
