# Supplementary material for: Pediatric celiac disease and coexisting immune-mediated conditions: a five-year single-center study from North-Eastern Romania
Source: Front Pediatr. 2026 Jun 3;14:1786392. doi: 10.3389/fped.2026.1786392 (PMC13272468; doi:10.3389/fped.2026.1786392)
Supplement: Supplementary file 1 [file Supplementaryfile1.docx]

Supplementary Material

**Table S1**. Shapiro–Wilk test for normality of continuous variables included in the statistical analyses

| Variable | Subgroup/Description | Shapiro-Wilk statistic | df | p-value |
| --- | --- | --- | --- | --- |
| Age at celiac disease diagnosis (years) | No immune-mediated condition | 0.847 | 40 | <0.001 |
|  | Immune-mediated condition present | 0.949 | 18 | 0.414 |
| Difference between age at T1DM and CD diagnosis (years) | Paired difference | 0.887 | 12 | 0.107 |
| Difference in fasting glucose (mg/dL) | Pre- vs post-GFD | 0.990 | 8 | 0.995 |
| Difference in HbA1c (%) | Pre- vs post-GFD | 0.977 | 8 | 0.945 |

**Note:**
Normality was assessed using the Shapiro–Wilk test. Detailed graphical assessments (Q–Q plots and boxplots) are provided in Supplementary Figures S1–S2.

**Abbreviations:**
CD, celiac disease; T1DM, type 1 diabetes mellitus; GFD, gluten-free diet; df, degrees of freedom.


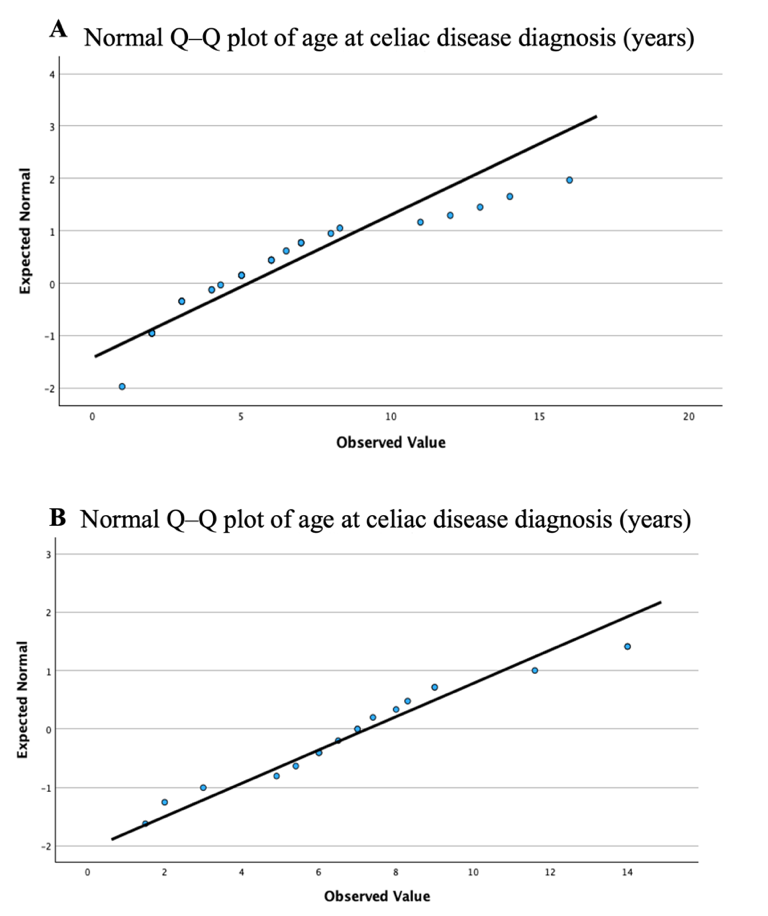


**Figure S1.** Normal Q–Q plots for age at celiac disease diagnosis according to immune-mediated condition status. (A) Immune-mediated condition absent. (B) Immune-mediated condition present.


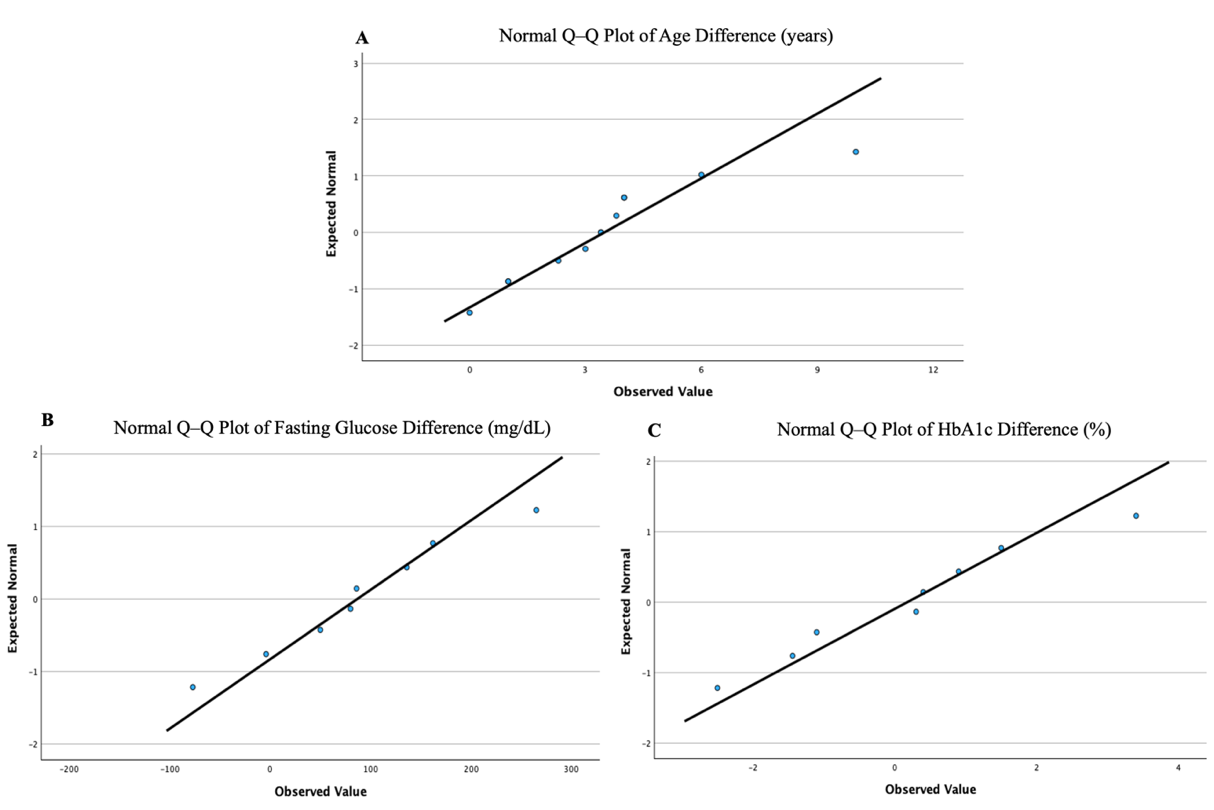


**Figure S2.** Normal Q–Q plots for paired differences in clinical variables. **(A)** Difference between age at diagnosis of type 1 diabetes mellitus and celiac disease. **(B)** Difference in fasting glucose levels before and after initiation of a gluten-free diet. **(C)** Difference in HbA1c levels before and after initiation of a gluten-free diet.

**Table S2.** Poisson regression model and goodness-of-fit statistics for annual counts of newly diagnosed celiac disease cases.

| **Goodness of Fit** | | | |
| --- | --- | --- | --- |
|  | Value | df | Value/df |
| Deviance | 11.851 | 3 | 3.950 |
| Scaled Deviance | 11.851 | 3 |  |
| Pearson Chi-Square | 13.031 | 3 | 4.344 |
| Scaled Pearson Chi-Square | 13.031 | 3 |  |
| Log Likelihood | -14.520 |  |  |
| Akaike's Information Criterion (AIC) | 33.041 |  |  |
| Finite Sample Corrected AIC (AICC) | 39.041 |  |  |
| Bayesian Information Criterion (BIC) | 32.259 |  |  |
| Consistent AIC (CAIC) | 34.259 |  |  |

**Notes:**
The dependent variable was the number of newly diagnosed celiac disease cases (count).
The model included an intercept and calendar year as predictor.
Information criteria (AIC, AICc, BIC, CAIC) are reported in smaller-is-better form and were computed using the full log-likelihood function.

**Table S3**. Negative binomial regression model and goodness-of-fit statistics for annual counts of newly diagnosed celiac disease cases.

| **Goodness of Fit** | | | |
| --- | --- | --- | --- |
|  | Value | df | Value/df |
| Deviance | 1.980 | 3 | .660 |
| Scaled Deviance | 1.980 | 3 |  |
| Pearson Chi-Square | 2.054 | 3 | .685 |
| Scaled Pearson Chi-Square | 2.054 | 3 |  |
| Log Likelihood | -14.453 |  |  |
| Akaike's Information Criterion (AIC) | 32.906 |  |  |
| Finite Sample Corrected AIC (AICC) | 38.906 |  |  |
| Bayesian Information Criterion (BIC) | 32.125 |  |  |
| Consistent AIC (CAIC) | 34.125 |  |  |

**Notes:**

The dependent variable was the number of newly diagnosed celiac disease cases (count).
The model included an intercept and calendar year as predictors.
Information criteria (AIC, AICc, BIC, CAIC) are reported in smaller-is-better form.
The full log-likelihood function was used to compute information criteria.
